# Knockout of MYOM1 in human cardiomyocytes leads to myocardial atrophy via impairing calcium homeostasis

**DOI:** 10.1111/jcmm.16268

**Published:** 2021-01-15

**Authors:** Chengwen Hang, Yuanxiu Song, Ya’nan Li, Siyao Zhang, Yun Chang, Rui Bai, Amina Saleem, Mengqi Jiang, Wenjing Lu, Feng Lan, Ming Cui

**Affiliations:** ^1^ Department of Cardiology Peking University Third Hospital Beijing China; ^2^ Beijing Lab for Cardiovascular Precision Medicine Anzhen Hospital Capital Medical University Beijing China

**Keywords:** calcium homeostasis, CaMKII, hESC‐CMs, myocardial atrophy, myocardial contraction, MYOM1 knockout, myomesin‐1 deficiency

## Abstract

Myomesin‐1 (encoded by MYOM1 gene) is expressed in almost all cross‐striated muscles, whose family (together with myomesin‐2 and myomesin‐3) helps to cross‐link adjacent myosin to form the M‐line in myofibrils. However, little is known about its biological function, causal relationship and mechanisms underlying the MYOM1‐related myopathies (especially in the heart). Regrettably, there is no MYMO1 knockout model for its study so far. A better and further understanding of MYOM1 biology is urgently needed. Here, we used CRISPR/Cas9 gene‐editing technology to establish an MYOM1 knockout human embryonic stem cell line (MYOM1*^−/−^* hESC), which was then differentiated into myomesin‐1 deficient cardiomyocytes (MYOM1*^−/−^* hESC‐CMs) in vitro. We found that myomesin‐1 plays an important role in sarcomere assembly, contractility regulation and cardiomyocytes development. Moreover, myomesin‐1‐deficient hESC‐CMs can recapitulate myocardial atrophy phenotype in vitro. Based on this model, not only the biological function of MYOM1, but also the aetiology, pathogenesis, and potential treatments of myocardial atrophy caused by myomesin‐1 deficiency can be studied.

## 1 INTRODUCTION

The sarcomere of cross‐striated muscle is composed of the Z‐disc at both ends and M‐band in the centre, which arranges thin and thick filaments in an orderly manner. Overlapping arrays of antiparallel myosin rods form the M‐band, and adjacent myosin rods are cross‐linked with each other through Myomesin gene family, becoming the M‐line.^1^ There are three members of the human Myomesin gene family, namely Myomesin‐1/Myomesin encoded by MYOM1, Myomesin‐2 / M‐protein encoded by MYOM2, and Myomesin‐3 encoded by MYOM3. MYOM1 expresses in almost all striated muscles, indicating its irreplaceability in striated muscles, whereas expression of MYOM2 and MYOM3 varies due to tissue, species, and developmental stage.^2^ Abnormalities in this family are considered to be a fundamental cause of myopathies, such as DCM,^3^, ^4^ HCM,^5^, ^6^ DM1,^7^ and LGMD2D.^8^ Among them, reports on myomesin‐1 and heart diseases are more common. EH‐myomesin is a splice variant of myomesin‐1, which was inserted with a flexible EH fragment in the middle of the protein, and it is mainly expressed in the embryonic heart.^9^ When it is abnormally expressed in the adult heart, it usually means the occurrence of heart diseases. EH‐myomesin was found up‐regulated in the early stage of DCM, and its expression level was positively correlated with ventricular dilatation, so it was once considered a biomarker for DCM.^3^ Missense mutation of the MYOM1 gene causes the dimerization affinity and thermal stability of Myomesin‐1 protein to decrease, which was involved in the progression of HCM.^6^ Moreover, when the exons of TTN which encodes the binding site of titin and myomesin‐1 were knocked out in the mouse heart, it caused the disintegration of myofibrils and cardiac atrophy.^10^ Up to now, most of these are correlational researches and there are no aetiology and pathology studies. The underlying mechanisms of MYOM1 mutation or alternative splicing causing cardiomyopathies are unclear.

Myomesin‐1 consists of 1685 amino acids and has 13 domains, including a unique N‐terminal domain, followed by two Ig‐like, five Fn‐like and five other Ig‐like domains.^11^ It behaves as a structural linker in sarcomere M‐line, connecting with muscle‐type creatine kinase (MCK), Titin and Obscurin family (Obscurin/OBSCN, Obscurin‐like‐1/OBSL1).^12^, ^13^, ^14^ Its N‐terminal domain is anchored to myosin. The C‐terminal domain dimerizes with each other to form an antiparallel dimer.^14^, ^15^ Using siRNA to inhibit the expression of MYOM1 in neonatal rat cardiomyocytes not only led to the failure of M‐band formation but also caused the disintegration of myofibrils, suggesting its important role in the assembly and stabilization of myofibrils.^16^ Besides, dimerized myomesin‐1 can reversibly stretch to 2.5 times its original length when exposed to low molecular stress, indicating it may act as a ‘shock absorber’ ‐a molecular spring similar to titin.^17^, ^18^ However, its specific process involved in the myofibrillar organization, mechanical sensing and the functions of its 13 domains is not transparent. Compared with another elastic protein‐titin, which has been studied widely in recent years, researches of myomesin‐1 have been largely ignored. Despite one knockdown study of MYOM1,^16^ there is no MYOM1 knockout model for genotype‐phenotype relations and detailed biological function study. The knockdown study used neonatal rat cardiomyocytes, but there are species differences between animal and human cardiomyocytes.

Now we have human embryonic stem cells (hESCs), which can be differentiated into cardiomyocytes (hESC‐CMs), skeletal muscle cells, etc, providing us with useful tools for studying human striated muscle diseases, without species restrictions. Thus, we generated an MYOM1*^−/−^* hESCs (H9) cell line with CRISPR/Cas9 system, which can be continuously subcultured, and myocytes differentiated from it provide a useful model to study biological functions of MYOM1 and the pathological mechanisms underlying MYOM1‐related myopathies. Here, wild type (WT) and MYOM1*^‐/‐^* hESCs (KO) were differentiated into cardiomyocytes and we found that myomesin‐1‐deficient cardiomyocytes present morphological abnormalities, myocardial dysfunction and atrophic remodelling. Disturbance of calcium homeostasis is probably the leading cause of myocardial dysfunction in KO CMs, especially due to the subtype transformation of CaMKII (decreased CaMKIIδ, but increased CaMKIIγ). Furthermore, we discovered that myocardial atrophy may be involved in increased expression of MuRF1, which can be positioned in M‐band and promote protein degradation.

## 2 MATERIALS AND METHODS

### 2.1 Cardiac differentiation of hESCs

Human embryonic stem cells H9 and mutant cells derived from it were kept in feeder‐free Matrigel plates (Corning) and maintained with PSCeasy medium (Cellapy) daily. Cells were passaged using 0.5 mmol/L calcium and magnesium‐free EDTA solution (HyClone) based on cell status and confluence. Human embryonic stem cells were induced to differentiate into cardiomyocytes by using chemically defined small molecules which can modulate Wnt/β‐catenin pathway.^19^, ^20^ Biochemical differences in glucose and lactate metabolism between cardiomyocytes and non‐cardiomyocytes was exploited for purification of hESC‐CMs.^21^, ^22^ All cells were cultured at 37°C, 5% CO_2_ humidified incubators.

### 2.2 Generation of MYOM1*^−/−^* hESC‐CMs

MYOM1 knockout cardiomyocytes were differentiated from genetically edited hESCs, in other words from MYOM1*^−/−^* hESCs. Single guide RNA (CTGGGCTATATAAGCAGCAG) was designed targeting MYOM1 by using online ‘CRISPR RGEN TOOLs’ (http://rgenome.net). Then, the sgRNA was ligated into the plasmid vector epiCRISPR for next cell transfection.^23^ 1.5 × 10^6^ dissociated cells from the H9 cell line were electroporated with electroporation mixed solution, containing 2.5 μg intact epiCRISPR plasmid and 100 μL electroporation buffer, by using the Human Stem Cell Nucleofection (Lonza). The electrotransformed cells were cultured in PSCeasy medium with Rho‐kinase inhibitor Y‐27632 to promote cell survival on the first day and then sieved with 0.3 µg/mL puromycin for 5‐7 days. Positive clones were picked out for gene sequencing verification to identify MYOM1*^‐/‐^* hESCs lines.

### 2.3 Immunofluorescence staining

Cell slides were fixed with 4% paraformaldehyde at room temperature for 15 minutes. After washing 3 times with PBS, cell membranes were permeabilized with 0.5% Triton X‐100 (Sigma) for 15 minutes, and then blocked with 3% bovine serum albumin (Sigma) for half an hour. Use the primary antibody to incubate cells at 4ºC overnight and wash them with PBS again. Then, cells were incubated in secondary antibodies without light for 1‐2 hours at room temperature. After washing 3 times with PBS for 5 minutes each, cell slides were counterstained for 15 minutes by using a DAPI‐containing antifade solution (Invitrogen). Fluorescence pictures were taken under a Laser Scanning Confocal Microscope (Lycra) and analysed with ImageJ software. Both primary and secondary antibodies were shown in Table S1.

### 2.4 Flow cytometry

Single cells were prepared according to a two‐step protocol using CardioEasy CM dissociation buffer I and II (Cellapy) and fixed with chilled fixation buffer (BD Biosciences) for 15 minutes at room temperature. Cell permeabilization and blocking were performed as described above in immunofluorescence staining. Cells were then stained by successive incubation of the primary and secondary antibodies for 30 minutes each. After washing and resuspending with PBS, the stained cells were measured by Flow Cytometer (EPICS XL, Beckman). Analysis and quantification were carried out with FlowJo and CytExpert software.

### 2.5 RNA isolation and quantitative real‐time PCR (qRT‐PCR)

Total RNA was extracted from about 1 × 10^6^ cells using the TRIzol^TM^ Reagent (Life Technologies) following the manufacturer's manual and dissolved with RNase‐free Wate (Qiagen). Further purification of RNA was achieved using DNase I (Life Technologies) and RNeasy Mini Kit (QIAGEN).RNA concentration was measured with the NanoDrop‐1000 spectrophotometer (Thermo Scientific). cDNA was synthesized using PrimeScript^TM^ reverse transcription system (Takara) according to the manufacturer's instructions. Continuously qRT‐PCR was performed using 2 × SYBR Master Mix (Takara) on the iCycler iQ5 (Bio‐Rad). Relative mRNA expression analyses were performed using △△C_T_ Method. Primer sequences used for qPCR were presented in Table S2.

### 2.6 Western Blot

Preparing cell lysis buffer for protein extraction, consisting of Mammalian Protein Extraction Reagent (Thermo, #78501),5 mmol/L EDTA (Thermo, #1861275), protease inhibitor cocktail (TERMO, #1861278) and phosphatase inhibitor cocktail (TERMO, #1862495). Cell lysis buffer was added to cell samples and placed on ice for 30 minutes, vibrating for 15 seconds every ten minutes to fully lyse the cells. The cell lysate was then centrifuged at 12700 g for 15 minutes at 4°C, and the resulting supernatant was evaluated for concentration by Pierce™ BCA Protein Assay Kit (Thermo, #23227). Heat‐denatured proteins are separated by SDS‐PAGE and transferred to PVDF membranes exploiting Mini‐Protean Tetra Cell (Bio‐Rad). Blocking was performed with QuickBlock™ Blocking Buffer (Beyotime) for 10 minutes, followed by separate incubation with primary and secondary antibodies. Both primary and secondary antibodies were shown in Table S1.

### 2.7 Ca^2+^ imaging

hESC‐CMs were singularized and seeded into a confocal dish or chamber (Sigma). Fluo‐4 AM (Beyotime) mother liquor was diluted to 5 µmol/L working solution with PBS for next cell loading. After washing three times with PBS, the cells were incubated with Fluo‐4 AM working solution at 37°C for 15 minutes during which 0.02% Pluronic F‐127 (Invitrogen) can be added to promotes Fluo‐4 AM into cardiomyocytes. Then, the cells were washed three times with PBS, and the Fluo‐4 fluorescence intensity was measured by a laser confocal microscope (Leica, TCS5 SP5, Germany) to determine the intracellular Ca^2+^ changes. The total scan time is 32.768 seconds, collecting four pictures, each for 8.192 seconds. Subsequently, a caffeine release experiment was performed using calcium‐free Tyrodes solution (Solarbio, China) to deplete Ca^2+^ in the extracellular fluid and 10 mmol/L caffeine to induce sarcoplasmic reticulum Ca^2+^ release. Data analysis was performed by ImageJ and Igor software.

### 2.8 RNA‐sequencing (RNA‐seq) Assay

Purified D30 cardiomyocytes were used for transcriptome sequencing and mRNA library establishment. After total RNA extraction, RNase H and DNase I were used for RNA purification. The purified RNA was then fragmented into pieces, followed by first‐strand and second‐strand cDNA synthesis. Afterwards, the double‐stranded DNA was purified by magnetic beads and amplified using PCR. The PCR products were denatured and circularized into single‐strand circle DNA (ssCir DNA), becoming the ultimate library after further amplification. Qualified library was analysed by the BGISEQ500 platform with a single‐end 50 bases reads (SE50). Clean reads were acquired after trimming reads with low quality, contamination or high levels of unknown base. These clean reads were then aligned to a reference genome sequence using Bowtie2 and HISAT2.

More significant and credible differentially expressed genes (DEGs) were screened out based on |log2 Fold change|≥1 and adjusted *P*‐value (FDR, *Q*‐value) <.001. Gene Ontology and KEGG pathway enrichment analysis of these DEGs were performed by Phyper under Hypergeometric test (https://en.wikipedia.org/wiki/Hypergeometric_distribution). Q‐value of terms or pathways less than 0.05 was significantly enriched. Bubble charts, histograms and heatmaps were drawn by R language package and online Omicshare Tools (https://www.omicshare.com/tools/). GSEA software (https://www.broadinstitute.org/gsea/) was used for Gene Set Enrichment Analysis with the following parameters: permutation type = geneset,metric = Signal2Noise,enrichmentstatistic = weighted,permutation = 1000. Significantly enriched gene sets were identified based on:|NES|≥1, *P*‐value < 5% and q‐value < 25%.

### 2.9 Cell proliferation and death detection

Cell proliferation ability was tested using the BeyoClick™ EDU‐594 assay kit (Beyotime) according to the manufacturer's instructions. Simply put, 10 μmol/L EDU solution was added to the culture medium, incubating for 18‐24 hours in the incubators. Labelled cells were fixed with 4% PFA at room temperature for 15 minutes. After washing three times with PBS, cells were permeabilized with 0.5% Triton X‐100 for 15 minutes. Cells were then stained with the click reaction solution containing Azide 594 for 30 minutes in the dark for fluorescence detection. Annexin V‐FITC/PI Apoptosis Detection Kit (Yeasen) was used to determine cell death. Cardiomyocytes were disassociated, resuspended and centrifuged at 300 *g* for 5 minutes. After that, cardiomyocytes were washed twice with pre‐cold PBS, and then, a 100 μL binding buffer was used to resuspend CMs. Next, 5 μL Annexin V‐FITC together with 10 μL PI staining solution was added and placed at room temperature for 10‐15 minutes. Finally, an extra 400 μL binding buffer was supplied and mixed thoroughly for flow cytometry.

### 2.10 Electron microscopy

Sheet‐shaped cardiomyocytes were fixed with 2.5% glutaraldehyde fixative solution (2.5% glutaraldehyde and 1% paraformaldehyde in PBS solution at pH 7.4). Then, osmic acid (containing 0.1 M sodium cacodylate, 2% osmium tetraoxide and 0.8% potassium ferrocyanide) was used for further immobilization. After dehydration with acetone, samples were embedded in Spurr resin and ultra‐thin sectioned. Subsequently, contrast staining was carried out with uranyl acetate and lead citrate. Image acquisition was made under a 120 KV transmission electron microscope equipped with a high‐resolution camera. Several myocardial fibres in each sample were randomly selected for observation, and typical structures were photographed under 6800x.

### 2.11 Statistical analysis

All data are presented as means ± SD. Statistical test methods mainly include Student t test between two groups or one‐way ANOVA with Tukey post‐test among multiple groups. The value of *P* < .05 was regarded as statistically significant (*), with**P < .01, ***P < .001, ****P < .0001.

## 3 RESULTS

### 3.1 Deletion of MYOM1 in hESCs lead to myomesin‐1‐deficient hESC‐CMs without affecting myocardial differentiation

**FIGURE 1:**
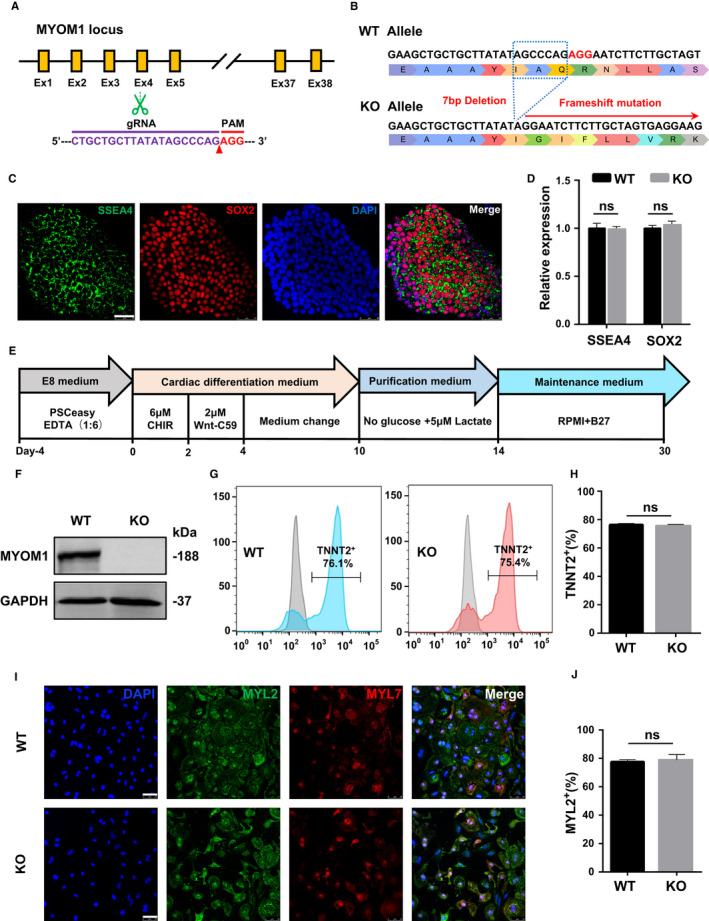
MYOM1^−/−^ hESCs have normal pluripotency and ability to differentiate into MYOM1^−/−^ hESC‐CMs. (A) Schematic illustration of human myomesin‐1’s total exons and the guide RNA designed for CRISPR/Cas9 mutagenesis. (B) Excision of 7 nucleotides resulting in frameshift mutation of whole protein from exon‐4. (C,D) Immunostaining and mRNA level of MYOM1^−/−^ hESCs for the pluripotency markers (Scalebar, 50 μm). (E) Schematic representation of small molecule‐based cardiac differentiation. (F) Western blot of MYOM1 protein and GAPDH from WT and KO CMs at day 20. (G,H) Flow cytometry of day 20 cardiomyocytes from two groups stained with troponin T (TNNT2/cTNT) without purification. (I,J) Immunostaining of day 20 CMs with MLC2v(MYL2) and MLC2a (MYL7) (Scalebar, 50 μm). Data are analysed with two‐sample *t* test and shown as means ± SD. ns, not significant

To generate MYOM1 knockout cell lines, a sgRNA was designed targeting the fourth exon of MYOM1 gene (Figure 1A) in hESCs (H9) using the epiCRISPR/Cas9 gene‐editing system.^23^ After puromycin sieving, resistant clones were genotyped by DNA sequencing to pick out homozygous MYOM1 knockout cell lines. One of these MYOM1^−/−^ hESCs lines was chosen for use with 7bp deletion in the fourth exon (Figure S1A), resulting in frameshift mutation of MYOM1 gene during transcription and translation (Figure 1B) and thus leading to protein deletion.

We determined that the MYOM1^−/−^ hESCs showed similar ES‐cell‐like gene expression to H9 cells (Figure 1C,D; Figure S1B), and possessed normal clone morphology and ability to differentiate into three embryonic germ layers (Figure S1D,1E). Then, we used a small molecule‐based differentiation protocol to generate purified hESC‐CMs (WT) and MYOM1^−/−^ hESC‐CMs (KO) (Figure 1E). We confirmed the depletion of myomesin‐1 protein in MYOM1^−/−^ hESC‐CMs by Western blot and immunofluorescence (Figure 1F, S1C). As Myomesin‐1 is mainly expressed in the M‐band of cardiomyocytes as a cytoskeleton protein, not in stem cells, we speculated MYOM1 knockout does not affect the differentiation efficiency of cardiomyocytes. Flow cytometry detection of myocardial‐specific marker cTNT (cardiac Troponin T) on cells after 15 days of differentiation showed that cTNT‐positive cells in the WT and KO groups were 76.1% and 75.4%, respectively, with no statistical difference (Figure 1G,H). Furthermore, MYL2 (ventricular‐specific) and MYL7 (atrial‐specific) staining suggested that the proportion of MYL2‐positive cells in WT and KO CMs are both around 80% (Figure 1I,J). The above results indicate that MYOM1 deletion in hESCs does not significantly affect cell characteristics and cardiac differentiation.

### 3.2 Myomesin‐1 deficiency results in sarcomere disassembly and cell lessening

**FIGURE 2:**
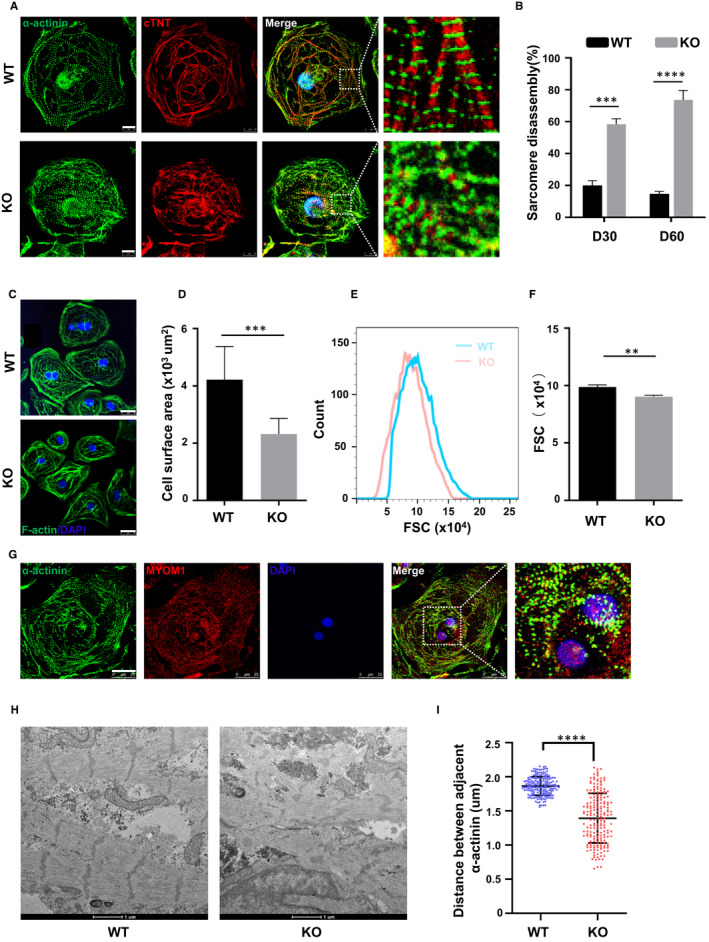
Knockout of MYOM1 in cardiomyocytes causes disordered sarcomere and smaller cells (A) Immunostaining of α‐actinin (green) and cTnT (red) shows sarcomere disassembly in day 30 KO CMs (Scale bar, 10 μm). (B) Quantification of cardiomyocytes exhibiting sarcomere disassembly from two groups, respectively, in day 30 and day 60 CMs(n = 150). (C,D) Image of cardiomyocytes stained with F‐actin/DAPI at day 30, and the mean cell area reduced in KO CMs (Scale bar, 25 μm). (E, F) Calibration of forward scatter (FSC) in flow cytometry (FSC;10 000 cells/sample) shows a decreased volume in day 30 MYOM1^−/−^ CMs (n = 3). (G) Image of myomesin‐1 locating in both cytoplasm and nucleus of day 30 WT CMs (Scale bar, 25 μm). (H) Disarranged sarcomere in knockout CMs observed by electron microscopy with partially twisted Z‐discs remaining (n = 185) (Scale bar, 1 μm). (I) Quantification of distances between adjacent α‐actinin, representing sarcomere length. Data are analysed with two‐sample *t* test and shown as means ± SD. **P < .01, ***P < .001, ****P < .0001

A previous study has shown that knockdown of Myomesin‐1 in primary neonatal rat cardiomyocytes leads to sarcomere disorders.^16^ We are concerned about whether this effect will be more pronounced in the MYOM1 knockout model. Therefore, we performed immunofluorescence staining on sarcomeric‐specific proteins ɑ‐actinin and cTNT, and found myomesin‐1‐deficient cardiomyocytes appeared a larger percentage of disrupted and disordered sarcomere relative to WT cardiomyocytes at day 30 (D30 CMs), with a more pronounced difference in D60 CMs (Figure 2A,B). A higher proportion of sarcomere disassembly with partially remaining Z‐discs in KO CMs was visualized by electron microscopy, which was in line with the above results (Figure 2H). As ɑ‐actinin is the marker of the Z‐disc, we next measured the distance between adjacent ɑ‐actinin, representing the distance between neighbouring Z‐discs, which also means the sarcomere length. KO CMs showed decreased and non‐uniform sarcomere length at day 30 (Figure 2A,H,I).

Interestingly, when we stained the myocardium with a‐actinin and myomesin‐1, we discovered that myomesin‐1 was present in both the nucleus and cytoplasm of cardiomyocytes at D30 CMs. Unfortunately, its location in the cytoplasm was messy and there was no regular striped distribution of myomesin‐1 (Figure 2G). We observed that WT hESC‐CMs and KO hESC‐CMs appeared to differ in size through an ordinary light microscope and confocal microscope after sarcomere staining. Then, we stained the complete cytoskeletal filamentous actin (F‐actin) with phalloidin as shown in Figure 2C, and found that the cross‐sectional area of KO hESC‐CMs showed significant reduction against WT hESC‐CMs (2321 ± 1160 vs 4220 ± 545.9 μm^2^, *P* < .05) (Figure 2D). The parameters FSC (forward scatter) of flow cytometry reflect the volume of cells to some degree. In flow cytometry, the FSC of KO hESC‐CMs is smaller than that of WT, statistically significant between groups (Figure 2E,F). These results suggest that MYOM1 knockout caused smaller CMs. As we have also generated other three homozygous clones, which were as follows: #2: −2 bp; #7: +1bp; #12: −5 bp (Figure S7A). We wanted to determine whether these results are reproducible from clone to clone; therefore, the #2 clone with 2 base pairs deletion was chosen for use. MYOM1‐deficient cardiomyocytes from this homozygous clone were equally smaller with disordered sarcomeres at day 30 (Figure S7B, S7C). In general, these findings demonstrate that MYOM1 affects the assembly of myofibrils and participates in maintaining cell size in cardiomyocytes, whereas MYOM1 knockout will cause changes in cardiomyocyte morphology.

### 3.3 MYOM1 knockout cardiomyocytes present impaired contractile properties

**FIGURE 3:**
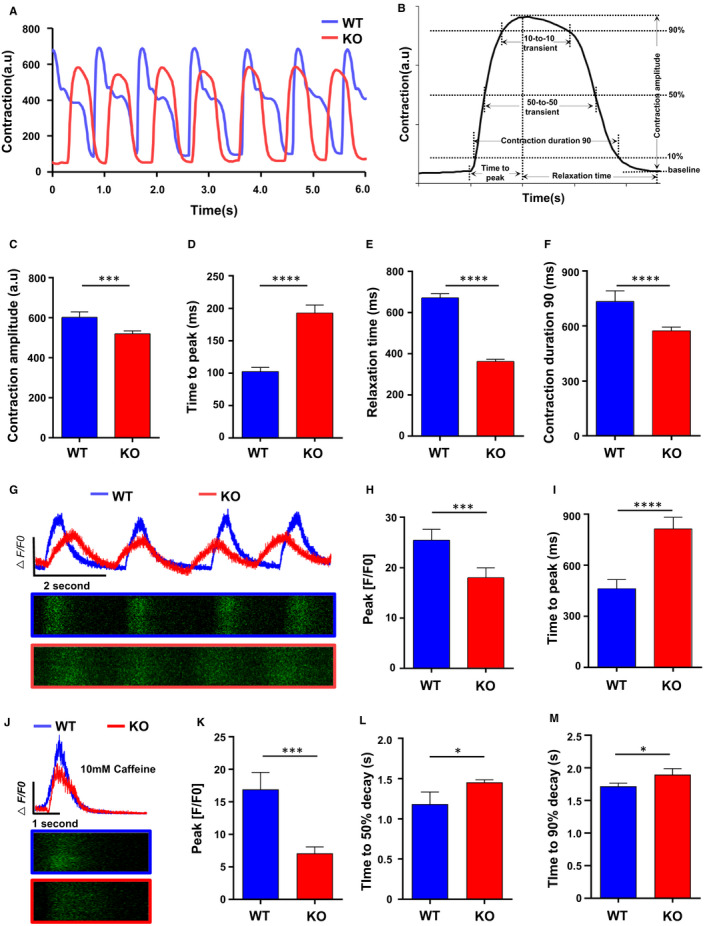
Myomesin‐1‐deficient human cardiomyocytes develop contractile dysfunction. (A) After video recorded, the curve of contraction versus time was output by ‘MUSCLEMOTION’, showing the beating activity of D30 WT and KO CMs. (B) Several parameters can be extracted from the above curve for analysis. (C) contraction amplitude, (D) time to peak, (E) relaxation time and (F) contraction duration 90 were measured. (G‐I) Ca^2+^ transients of day 30 cardiomyocytes were imaged in individual cells after loaded with 5 μmol/L Fluo‐4AM (n = 20 cells per group). (J‐M) Ca^2+^ transient induced by 10 mmol/L caffeine exposure between WT and KO CMs (n = 5 cells per group), and relevant parameters were measured. Data are analysed with two‐sample t test and shown as means ± SD. *P < .05, ***P < .001, ****P < .0001

Christine L, et al^24^ developed a software tool ‘MUSCLEMOTION’, which can measure the contractility of cardiomyocytes in vivo and in vitro by just taking a video of beating cardiomyocytes, with accurate outcomes consistent with current gold standards for contraction measurement. The main principle is using a mathematical algorithm model to analyse the changes in pixels at a certain point during cardiomyocytes beating, and then convert them into related parameters of myocardial contraction and relaxation, including contraction amplitude, contraction duration90, and relaxation time (Figure 3A‐3B). In the following experiments, we used the above software to analyse videos of WT and KO hESC‐CMs. We unveiled significantly decreased contraction duration, relaxation time and contraction amplitude in KO hESC‐CMs, but accompanied by increased time to peak (Figure 3A, 3C‐3F). Similar results were output using another improved algorithm ‘MYOCYTER’.^25^ As calcium homoeostasis is closely related to the contraction behaviour of cardiomyocytes, we further validated the above results through Ca^2+^ transient detection. Amplitude and time course of Ca^2+^ transients of Fluo‐4 AM loaded cardiomyocytes were recorded by line‐scanning confocal microscopy. As shown in Figure 3G‐3I, the amplitude of Ca^2+^ transients in knockout CMs decreased, but the rising phase (time to peak) of Ca^2+^ transients increased, which was consistent with the above video results. Besides, when analysing Ca^2+^ transients throughout the whole cardiomyocyte by video recording (not on a particular line), we also observed a decrease in the amplitude of calcium transients (Figure S2A). Ulteriorly, we explored whether there is any difference in stored Ca^2+^ in sarcoplasmic reticulum (SR) between two groups. Accordingly, we estimated SR Ca^2+^ load by caffeine‐induced Ca^2+^ release and found that the abundance of SR Ca^2+^ in MYOM1 KO myocytes is lower than that in WT myocytes (Figure 3J, K). However, the decay phases of stored Ca^2+^ release were slower in KO CMs (Figure 3L, M).

**FIGURE 4:**
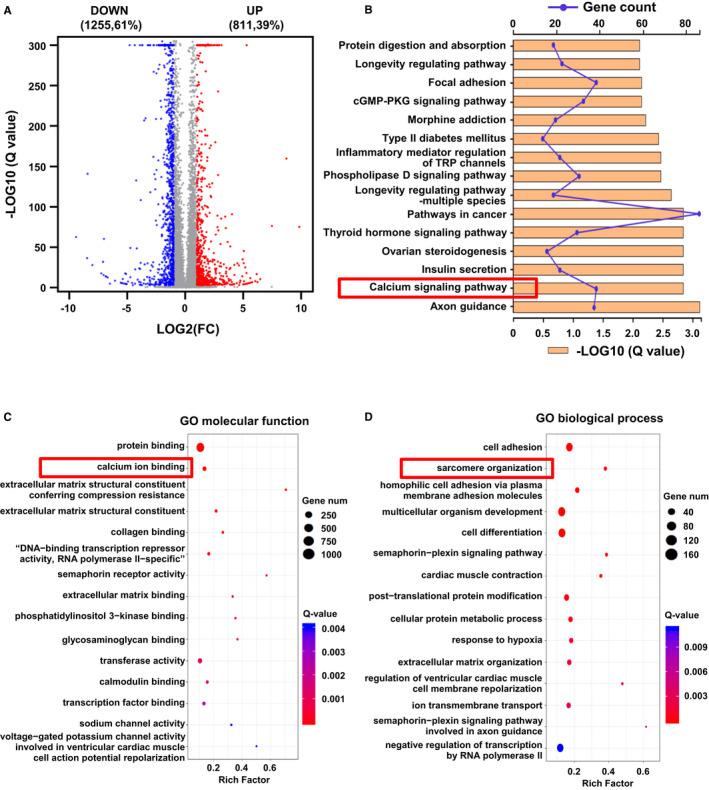
Transcriptomes are different between myomesin‐deficient and wild‐type CMs. (A) Gene expression profile was examined by RNA‐seq. The volcano plot shows differentially expressed genes between two groups (n = 3, respectively). Red denotes up‐regulated genes, whereas blue represents down‐regulated genes. (B) Significantly enriched KEGG (Kyoto Encyclopedia Genes and Genomes) pathways in differential genes. The Ca^2+^ signalling pathway enriched the second most differential genes with high reliability. (C,D) GO analyses include three categories, but only molecular function and biological process are shown here

Moreover, we used different concentrations of Phenylephrine (PE), an α receptor agonist to stimulate the contraction of myocardial cells. We found that in KO cells, 50 μmol/L or a higher dose of PE cannot further increase myocardial contractility. In contrast, WT cells have a continuous increase in contractility under the concentration from 25 μmol/L to 100 μmol/L (Figure S2B, 2C). Similar results were also observed when using Isoprenaline (ISO), a β receptor agonist (Figure S2D, 2E). Taking together the above results, we confirmed that myomesin‐1‐deficient hESC‐CMs develop contractile dysfunction.

### 3.4 MYOM1 knockout cardiomyocytes underwent molecular biology changes

**FIGURE 5:**
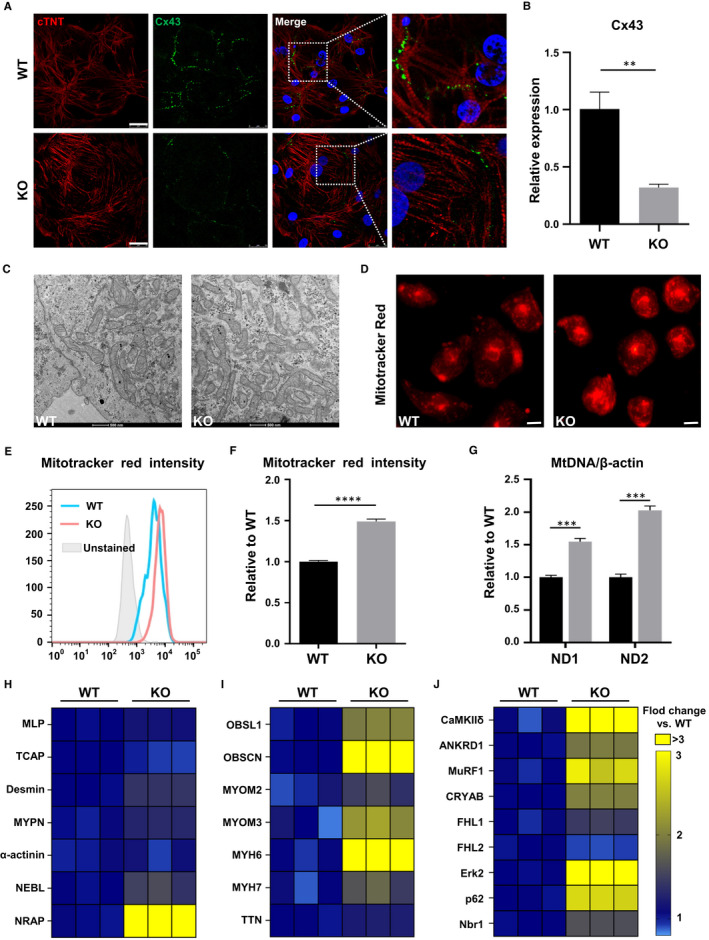
MYOM1^−/−^ hESC‐CMs are bearing many molecular and biological changes. (A) Confocal images of Cx43 and cTNT show an obvious decrease of Cx43 in D30 KO CMs compared with WT CMs (Scale bar, 25 μm). (B) Quantification of Cx43 on RNA level. (C) Representative transmission electron micrographs showing increased mitochondria in D30 KO CMs (Scale bar, 500 nm). (D, E, F) Images and fluorescence intensity of Mitotracker red, which stained mitochondria, also reveal an increase in the number of mitochondria in KO CMs (Scale bar, 50 μm). (G) Likewise, the ratio of mitochondrial DNA (ND1 and ND2) to nuclear DNA(β‐actin) elevated in MYOM1 knockout CMs. (H‐J) Respective heatmap of Z‐discs‐related, M‐band‐related and M‐band signalling associated genes expression in WT and KO CMs. Data are analysed with two‐sample t test and shown as means ± SD. **P < .01, ***P < .001, ****P < .0001

The important roles of mitochondria and Cx43 in cardiovascular diseases had been reviewed previously.^26^ In the following research, we focused on these. Our cardiomyocytes are mainly ventricular myocytes, and connexin 43 (Cx43) is the main gap junction protein in ventricular cardiomyocytes, helping to propagate electrical signal and synchronize cardiac muscle contraction.^27^ Its abundance and distribution are essential for maintaining the health of myocardial cells.^28^ Therefore, we compared Cx43 of the two groups and found that in the MYOM1 knockout CMs the abundance of Cx43 was significantly reduced, which is consistent with the results of qPCR (Figure 5A,B). But its subcellular localization was not affected by MYOM1 knockout (Figure 5A).

In our daily process of cell culture, we found that compared with WT cardiomyocytes, the medium of KO cardiomyocytes changes from red to yellow more quickly (Figure S5A). Based on this, we speculate that the lack of MYOM1 will affect cell metabolism. It is known that mitochondria are the main source of energy for myocardial cells, which are distributed widely in cardiomyocytes and provide ATP for myocardial contraction and metabolism. Therefore, we investigated the effect of MYOM1 knockout on mitochondria of cardiomyocytes. First, through electron microscope observation, we found that the number of mitochondria in KO cardiomyocytes was more than that in WT cardiomyocytes under the same magnification (Figure 5C). At the same time, the cross‐sectional area of mitochondria showed no difference between the two groups (Figure S5E). Accordingly, we used Mitotracker Red to stain mitochondria; more mitochondria were observed in KO CMs (Figure 5D, S5B). Next, the mitochondrial content in KO and WT CMs was quantified by comparing the ratio of mitochondrial‐specific ND1 and ND2 genes with nuclear housekeeping gene ACTB (encoding β‐actin). We found that the mtDNA/nDNA ratio showed a substantial increment in KO CMs (Figure 5G). Finally, we confirmed the increase of mitochondria in myomesin‐1 deficient cardiomyocytes by flow cytometry after Mitotracker staining (Figure 5E,F). Furthermore, cardiomyocytes were stained with MitoSOX Red to investigate the mitochondrial reactive oxygen species (ROS), which is related to cell apoptosis. There were no significant changes in the level of mitochondrial reactive oxygen species (ROS) in KO CMs when compared with WT CMs (Figure S5C, 5D, 5F).

Besides, we examined the molecular changes involved in sarcomere assembly. As shown in Figure 5H, molecules involved in Z‐disc formation did not change significantly except NRAP, whereas related molecules constituting the M‐band switched significantly, in which OBSL1 and OBSCN were significantly increased (Figure 5I). To further dissect the molecular basis between phenotypes, we evaluated the RNA level of several proteins involved in the hypertrophy/atrophy pathway at M‐band according to previous reports.^10^, ^29^ Among them, CaMKIIδ was significantly down‐regulated which is related to the calcium pathway, whereas MuRF1 participating in protein degradation was significantly elevated in KO CMs (Figure 5J).

### 3.5 Different transcriptome between WT and KO hESC‐CMs

**FIGURE 6:**
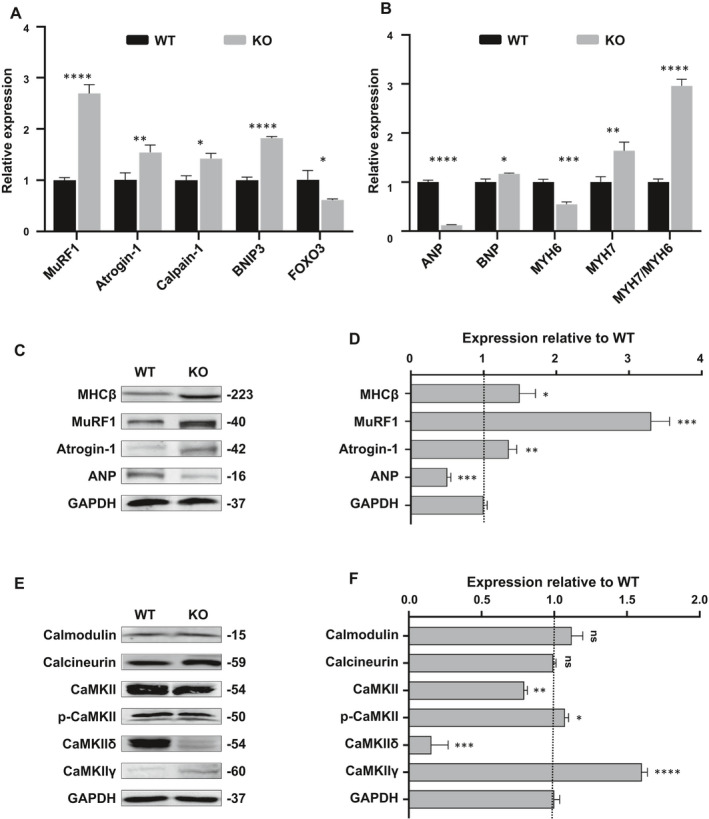
Myomesin‐1‐deficient CMs display atrophic remodelling and abnormal CaMKII activity. (A) Quantitative real‐time polymerase chain reaction reveals that molecular markers of muscle atrophy significantly increased in KO CMs. (B) An atrophic switching of MHC isoform predominance occurred in KO CMs, namely decreased MYH6 but increased MYH7. ANP level showed a dramatic decline, whereas BNP increased slightly. (C, D) Western blot analysis of several important markers of myocardial atrophy, quantification of protein expression was normalized by GAPDH. (E, F) The expression of several major proteins in Ca^2+^‐dependent pathways was measured and normalized by GAPDH. Data are analysed with two‐sample *t* test and shown as means ± SD. *P < .05, **P < .01, ***P < .001, ****P < .0001

To investigate the biological functions of MYOM1 and the potential molecular mechanisms underlying MYOM1 knockout, we performed transcriptomic sequencing on D30 WT and KO cardiomyocytes. Approximately 2066 genes differ in expression between WT and KO CMs, 40% up‐regulated, and 60% are down‐regulated (Figure 4A, S4C). Then, we carried out KEGG pathway analysis to find the enrichment of differentially expressed genes in metabolic pathways. Calcium signalling pathway, the second enriched pathway, is more closely related to the biological function of the MYOM1 gene and became the focus of our research (Figure 4B). Moreover, after GO analysis of differential genes, we identified 27, 17, and 12 functional class differences in biological process, cell component, and molecular function (Figure S4A). Protein binding is the most significant category of molecular functions, which gives us clues that we need to pay attention to the proteins that bind to myomesin‐1 (Figure 4C). Also, the second‐ranked category ‘calcium ion binding’, once again suggests the role of Ca ^2+^ related pathways in the biological function of MYOM1 (Figure 4C). Just like previous studies reported that structural components of M‐band such as titin and obscurin, both have calmodulin (CaM) binding sites, and these two proteins are anchored in the M‐band by binding to myomesin‐1.^30^ Among the cell component, membrane enriched most differential genes, indicating that the sarcomere may be connected to the cell membrane through myomesin‐1 (Figure S4B). Simultaneously, cell adhesion and sarcomere organization are the top two categories in biological processes (Figure 4D). Taken together, these findings suggest that MYOM1 is involved in regulating biological processes (especially sarcomere assembly) and may play an essential role in calcium‐related pathways. We further studied two common Ca^2+^‐dependent pathways in cardiomyocytes: CaM‐calcineurin and CaM‐CaMKII (mediated by CaMKIIδ and CaMKIIγ in the heart) pathway.^31^ CaMKII can directly regulate the activity of SERCA2a and RYR2, which mediate the sarcoplasmic reticulum Ca^2+^ cycle,^32^ and it is involved in cardiac maladaptive remoulding. In general, CaMKIIδ is the dominant CaMKII isoform in the heart, while the expression of CaMKIIγ is relatively low. As shown in Figure 6E,F, calmodulin (CAM) and calcineurin were at the same level between the two groups, but CaMKII decreased in KO CMs, appearing as a dramatic decrease in CaMKIIδ, while a medium increase in CaMKIIγ. These findings are likely to be an important basis for revealing MYOM1‐deficient phenotype.

### 3.6 Myomesin‐1 knockout CMs can mimic the phenotype of myocardial atrophy in vitro

In the above experiments, we confirmed that myomesin‐1 deficiency causes a decrease in myocardial volume, so the first thing we thought of is the relationship between myomesin‐1 deficiency and myocardial atrophy. Therefore, we focused on the genes that can act as markers in the process of cardiac atrophy.^33^, ^34^, ^35^ Then, we detected ubiquitin ligase MuRF1 (TRIM63), ubiquitin ligase Atrogin‐1 (MAFbx), the neutral protease calpain‐1 (CAPN1), BCL2 interacting protein‐3 (BNIP3) and Fork head box O3 (FOXO3) by qPCR. The expression of most of these genes was increased in MYOM1 knockout CMs (Figure 6A), especially MuRF1 and Atrogin‐1, which was also confirmed by Western blot (Figure 6C,D). Interestingly, we also identified that there was a switch of MHC (myosin heavy chain) isoform in KO CMs, from MHC‐α (MYH6) to MHC‐β (MYH7), which is an important feature of atrophic remodelling (Figure 6B,C). The abundance of ANP was not increased in KO CMs as previously reported,^33^, ^36^ even there was a significant decrease (Figure 6B‐6D). In summary, we found that myomesin‐1 knockout CMs exhibit reduced cell volume, decreased contractility, together with a molecular signature of muscle atrophy, which can recapitulate myocardial atrophy phenotype in vitro.

## 4 DISCUSSION

Combining CRISPR/Cas9 editing system and stem cell differentiation technology, we produced an MYOM1 knockout human embryonic stem cell line and generated myomesin‐1‐deficient cardiomyocytes, which are a brand new loss‐of‐function model. This model lacks myomesin‐1 in cardiomyocytes, an important component of the sarcomeric M‐line. We illustrated that myomesin‐1 plays a role in cardiomyocytes with regard to sarcomere integrity, cell growth, cardiomyocyte contractility and calcium homoeostasis. Besides, our results suggest that myomesin‐1 is involved in the regulation of cardiac atrophic remodelling, which may be a consequence of changes in Ca^2+^ related pathway and MuRF1 up‐regulation .

Due to the lack of a functional loss model of myomesin‐1 in myocardial cells, the biological functions of myomesin‐1 and its important role in heart diseases have not yet been clarified. Previous studies mostly utilized non‐hereditary methods, including overexpression of peptides or down‐regulation by siRNA in murine myoblast or neonatal cardiomyocytes.^5^, ^16^, ^37^ However, species differences and difficulties in acquiring and culturing cardiomyocytes in vitro could make the researches complicated. Now, our MYOM1*^−/−^* hESCs can be passaged and expanded in vitro, and differentiated into cardiomyocytes, generating a human‐derived, long‐term cultured in vitro MYOM1‐deficient myocardial model. What's more, MYOM1^‐/‐^ hESCs can also be differentiated towards many types of cells to establish various loss‐of‐function models, such as skeletal muscle cells (SkMCs)^38^, ^39^, ^40^ and pancreatic islets.^41^, ^42^

Previous study showed that using siRNA to inhibit the expression of MYOM1 in neonatal rat cardiomyocytes (NRCs) resulted in mislocalization of obscurin, failure of M‐band formation and disassembly of sarcomere,^16^ but the sarcomere M‐band or Z‐disc was not directly shown in this study. Here, we have also proved that the maximum inhibition of MYOM1 (MYOM1 knockout) caused sarcomere disorder, and the expression level of obscurin was significantly increased. Besides, we directly showed electron micrographs of sarcomeres. However, there is no M‐band or M‐line observed by an electron microscope until one year after differentiation,^43^, ^44^ and it is believed that the appearance of M‐line is due to the up‐regulation of another gene MYOM2 along with cardiac maturation.^45^ Although MYOM2 and MYOM3 were increased in KO CMs relative to WT CMs, their absolute expression was still at a low level and there was still no M‐line in both groups. These indicate that the knockout of MYOM1 cannot cause compensatory replacement of myomesin‐1 by other family members. Our research provides more evidence for explaining different expression profiles of myomesin family members.^46^, ^47^ They cannot compensate for each other, and they are not mutually redundant. Even though there is no M‐line in our myocardium, clear Z‐disc structures can be distinguished in day 30 cardiomyocytes. Z‐discs of MYOM1^−/−^ hESC‐CMs were more deformed and twisted. Their arrangement was no longer regular, resulting in different sarcomere lengths. We did not find great changes in the expression profiles of Titin (connecting M‐line and Z‐disc) or Z‐disc‐related proteins, except obscurin which is mainly located in M‐band. We speculate that the effects of MYOM1 knockout on Z‐discs are probably due to the disruption of the cross‐linking between Obscurin, Obsl1 (combined with Titin and MYOM1), Titin and myomesin‐1, instead of molecular changes in Z‐discs.

Cyclic contraction and relaxation are the basic characteristics of cardiomyocytes. We are concerned about the role of MYOM1 in myocardial contraction and relaxation. We found that knockout of MYOM1 resulted in a decrease in the contractility of cardiomyocytes, but no occurrence of arrhythmia, regardless of under basal conditions or stressed conditions, indicating that the structural gene MYOM1 mainly affects myodynamia, but has little effect on myoelectricity. After detecting Cx43, which affects myocardial electrical conduction and contraction, we only found a decrease in its abundance, but no apparent abnormal subcellular localization. As previously reported, the reduction of Cx43 in muscles could prevent and protect myocardial and skeletal muscle damage during muscular dystrophy.^48^, ^49^ We believe that the decrease of Cx43 in myomesin‐1‐deficient cardiomyocytes is a protective mechanism to prevent the damage of myocardial atrophy caused by myomesin‐1 deficiency. But, we think that reduction of Cx43 in KO CMs may lead to a decrease in the coordination ability between cells, which in turn weaken myocardial contractility to some degree. While the decreased calcium transient amplitude and sarcoplasmic reticulum (SR) calcium storage in KO CMs may be the main reason for the reduced contractility. Increased mitochondria that provide more energy for myocardial contraction should be a compensatory change. When the myocardial contractility decreases, more mitochondria are needed to produce more energy to promote the contraction of cardiomyocytes. However, due to the structural abnormality of KO cardiomyocytes (primary sarcomere disassembly), the increase in mitochondrial energy supply to promote contractility cannot compensate for the decrease in cell contractility resulted from structural changes.

Previous data have shown that the increase of EH‐myomesin leads to DCM,^3^ while the abnormal function of mutant myomesin‐1 is involved in the process of HCM.^6^ Besides, the positive regulation of myomesin‐1‐mediated sarcomere organization can induce cardiac hypertrophy.^5^ Here, we discussed for the first time that MYOM1 knockout in CMs could cause sarcomere disorganization and atrophic remodelling. Michael, et al^10^ have reported that when specifically knocking out the gene encoding the binding site of titin and myomesin‐1 in the mouse heart, the mouse presents disordered myofibrils, decreased myocardial contractility, and cardiac atrophy. Our research has reproduced the phenotype resulted from knockout of titin M‐band region to some degree. Thus, we speculate that the abnormal connection between titin and myomesin‐1 in the M‐band may be an essential factor and potential intervention target for cardiac atrophy.

Cardiac atrophy is mainly measured by the mass of the heart. The main factors leading to the decline in cardiac mass are decreased cell volume or increased cell attrition. Researchers have reported that intact myomesin‐1 and its specific Fn‐like or Ig‐like domains can stimulate human myoblast proliferation.^37^To investigate the effect of MYOM1 knockout on the number of cardiomyocytes, we next tested the proliferation and apoptosis of cardiomyocytes. We chose hESC‐CMs at day 20 post‐differentiation for EDU staining and flow cytometry to detect cell proliferation. However, results showed that there was no significant statistical difference in the proliferation ability of WT and KO CMs (Figure S3A‐D). Furthermore, the apoptosis level of KO hESC‐CMs was also not significantly different from that of WT hESC‐CMs (Figure S3E‐G). Therefore, we believe that there was no prominent apoptosis and proliferation imbalance in KO CMs, which can lead to increased cell attrition. MuRF1 is considered to be a relatively specific marker of cardiac atrophy, which promotes protein degradation.^50^, ^51^ It is speculated that myocardial atrophy caused by myomesin‐1 deficiency is probably the result of the up‐regulation of MuRF1, which leads to disorders of protein degradation and synthesis, but this still needs further studies on protein metabolism. Moreover, studies have shown that MYOM1 can be expressed in the nucleus of cardiomyocytes of newborn puppies and rats, causing differential expression of other genes, and may play a potential role as a transcription factor.^5^, ^52^ Here, we further confirmed that MYOM1 can also be expressed in nucleus of immature human cardiomyocytes. Deletion of MYOM1 in cardiomyocytes resulted in failure of shutting down genes related to angiogenesis and epithelial‐mesenchymal transition (Figure S6A), but the predominance of genes related to cardiac septum morphogenesis and development was lost (Figure S6B). This is a very significant discovery and is worthy of in‐depth study.

Our research not only provides a beneficial human‐derived loss‐of‐function model for studying biological functions and pathogenic mechanisms of myomesin‐1, but also conducts extensive research on the phenotype caused by myomesin‐1 deficiency, and points out the CaMKII subtype transformation may be an important factor for the atrophic phenotype. However, the limitation of this study includes two aspects. On the one hand, the cardiomyocytes used are not mature enough, and their maturity is equivalent to embryonic myocardium differing from adult cardiomyocytes. On the other hand, this study is mainly a phenotypic investigation and lacks in‐depth analysis of mechanism and intervention measures.

## CONFLICT OF INTEREST

The authors declare that there is no conflict of interest concerning the experiments or this paper.

## AUTHOR CONTRIBUTION

**Chengwen Hang:** Data curation (lead); Formal analysis (lead); Investigation (lead); Writing‐original draft (lead). **Yuanxiu Song:** Data curation (equal); Formal analysis (equal); Methodology (equal). **Yanan Li:** Data curation (equal); Formal analysis (equal); Validation (equal). **Siyao Zhang:** Data curation (equal); Formal analysis (supporting); Visualization (equal). **Yun Chang:** Data curation (supporting); Formal analysis (equal); Visualization (equal). **Rui Bai:** Formal analysis (equal); Resources (equal); Visualization (supporting). **Amina Saleem:** Validation (equal); Writing‐original draft (supporting). **Mengqi Jiang:** Formal analysis (equal); Investigation (supporting); Resources (supporting). **Wen‐Jing Lu:** Conceptualization (equal); Methodology (equal); Validation (equal). **Feng Lan:** Project administration (lead); Validation (lead); Writing‐review & editing (lead). **Ming Cui:** Funding acquisition (lead); Project administration (lead); Supervision (lead).

## Supporting information

Figure S1

Figure S2

Figure S3

Figure S4

Figure S5

Figure S6

Figure S7

Table S1

Table S2

## Data Availability

The data that support the findings of this study are available from the corresponding author upon reasonable request.
