# Supplementary figures and images for: Knockout of MYOM1 in human cardiomyocytes leads to myocardial atrophy via impairing calcium homeostasis
Source: J Cell Mol Med. 2021 Jan 15;25(3):1661–76. doi: 10.1111/jcmm.16268 (PMC7875908; doi:10.1111/jcmm.16268)

FIGURE S1

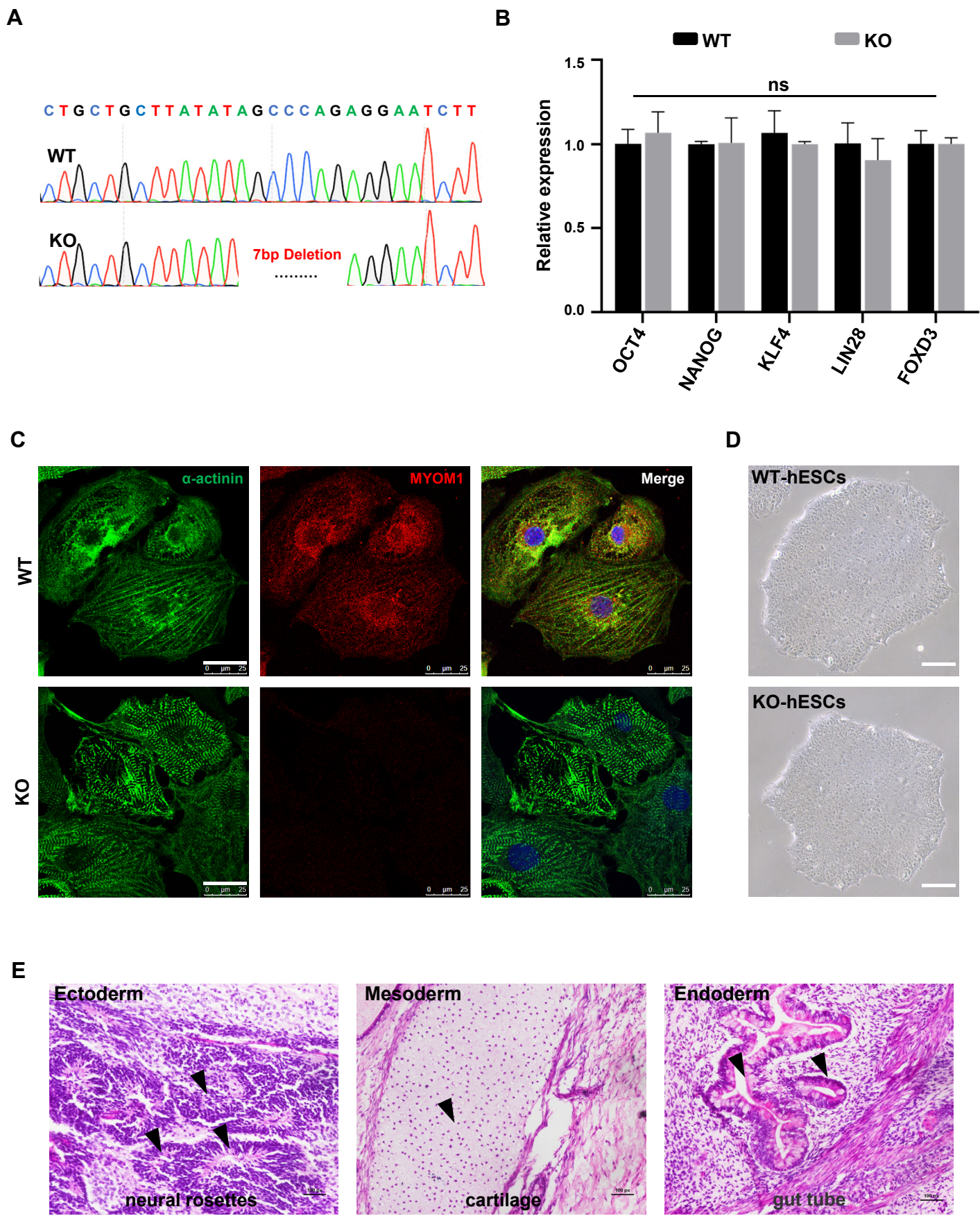

Supplement: Supplementary file 1 — Figure S1 [file JCMM-25-1661-s001.pdf]

FIGURE S2

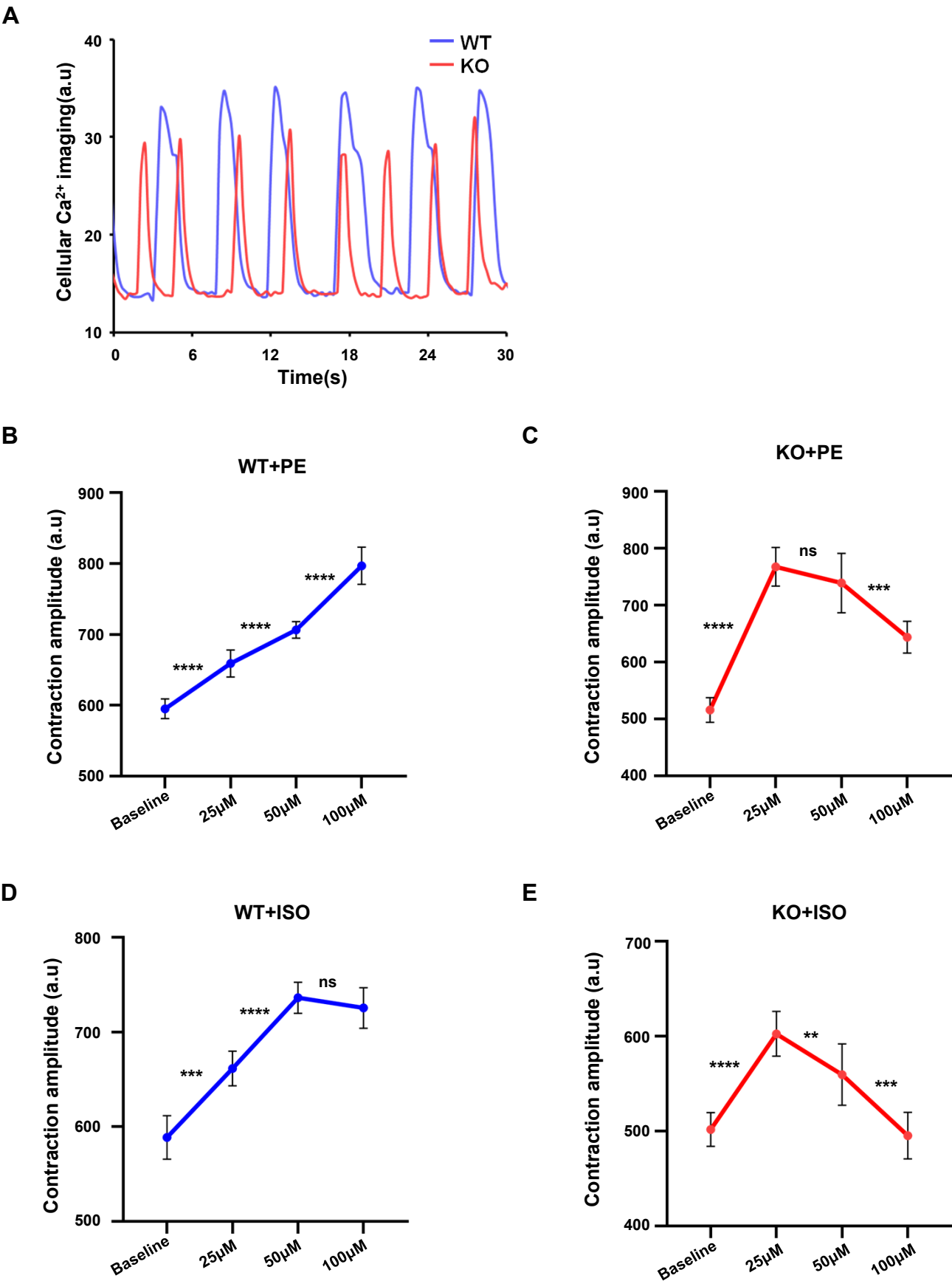

Supplement: Supplementary file 2 — Figure S2 [file JCMM-25-1661-s002.pdf]

FIGURE S3

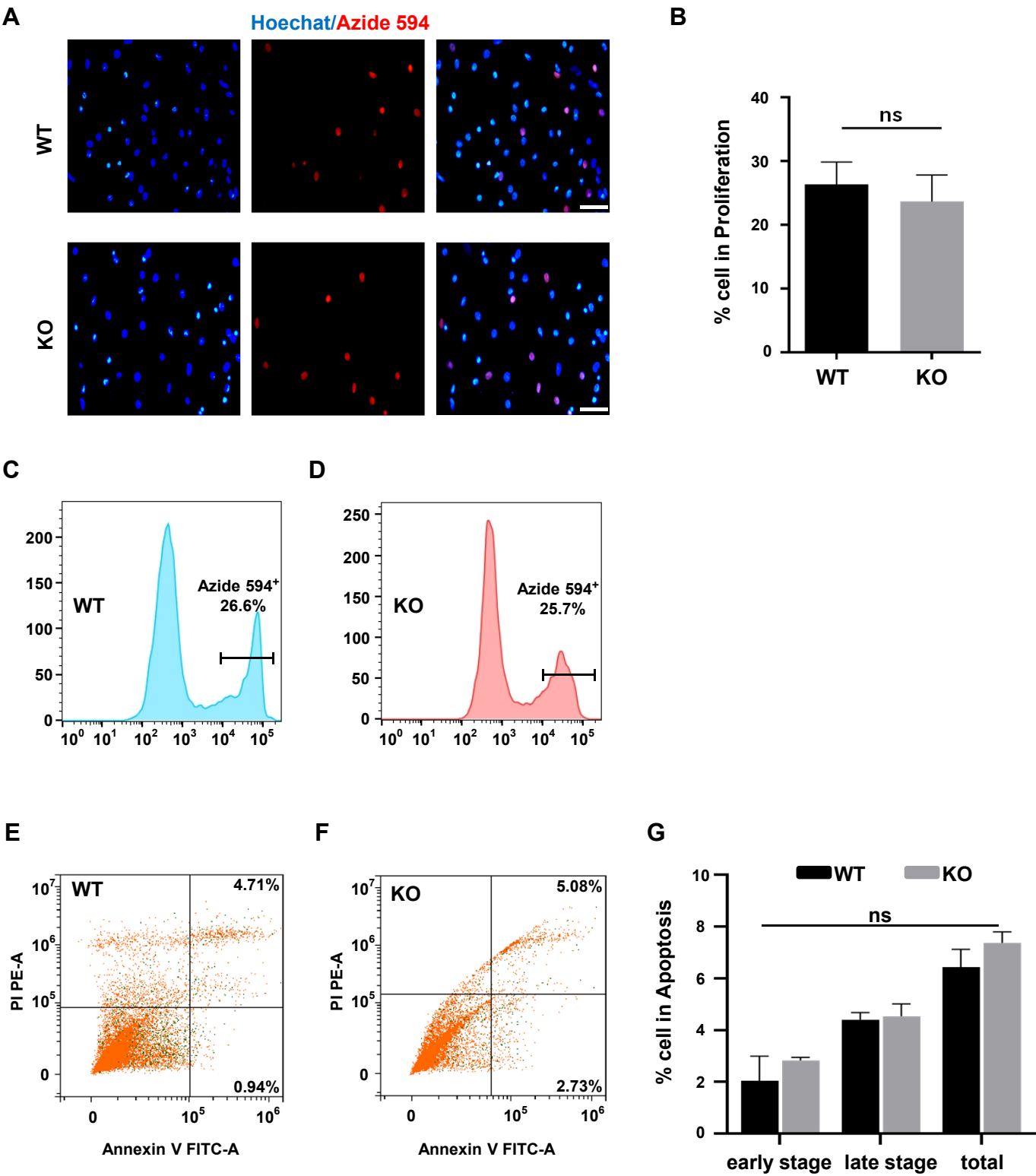

Supplement: Supplementary file 3 — Figure S3 [file JCMM-25-1661-s003.pdf]

FIGURE S4

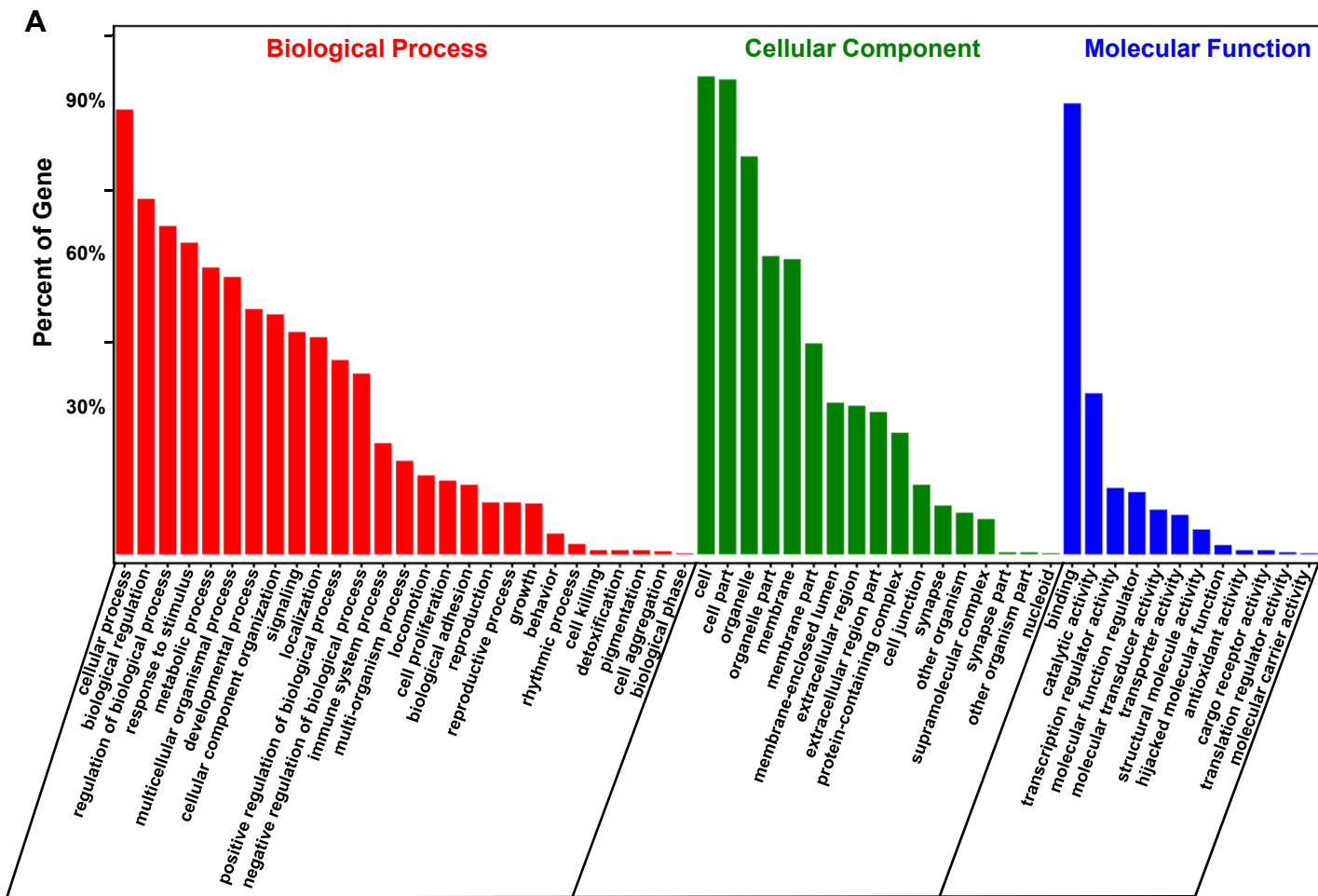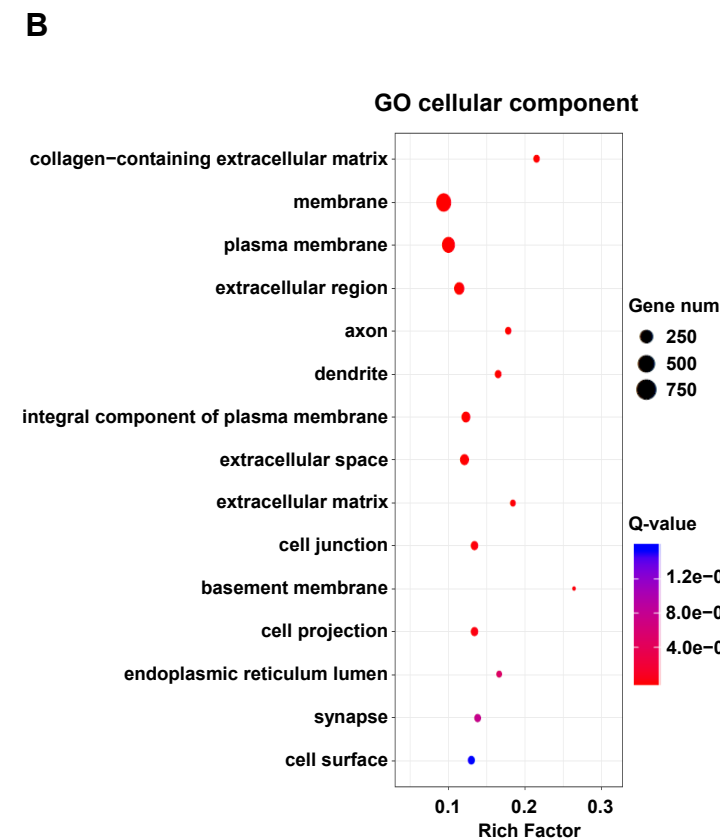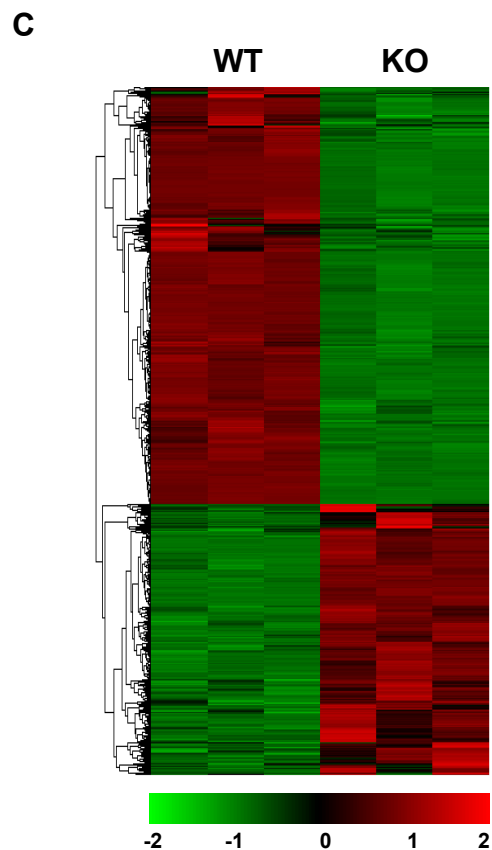

Supplement: Supplementary file 4 — Figure S4 [file JCMM-25-1661-s004.pdf]

FIGURE S5

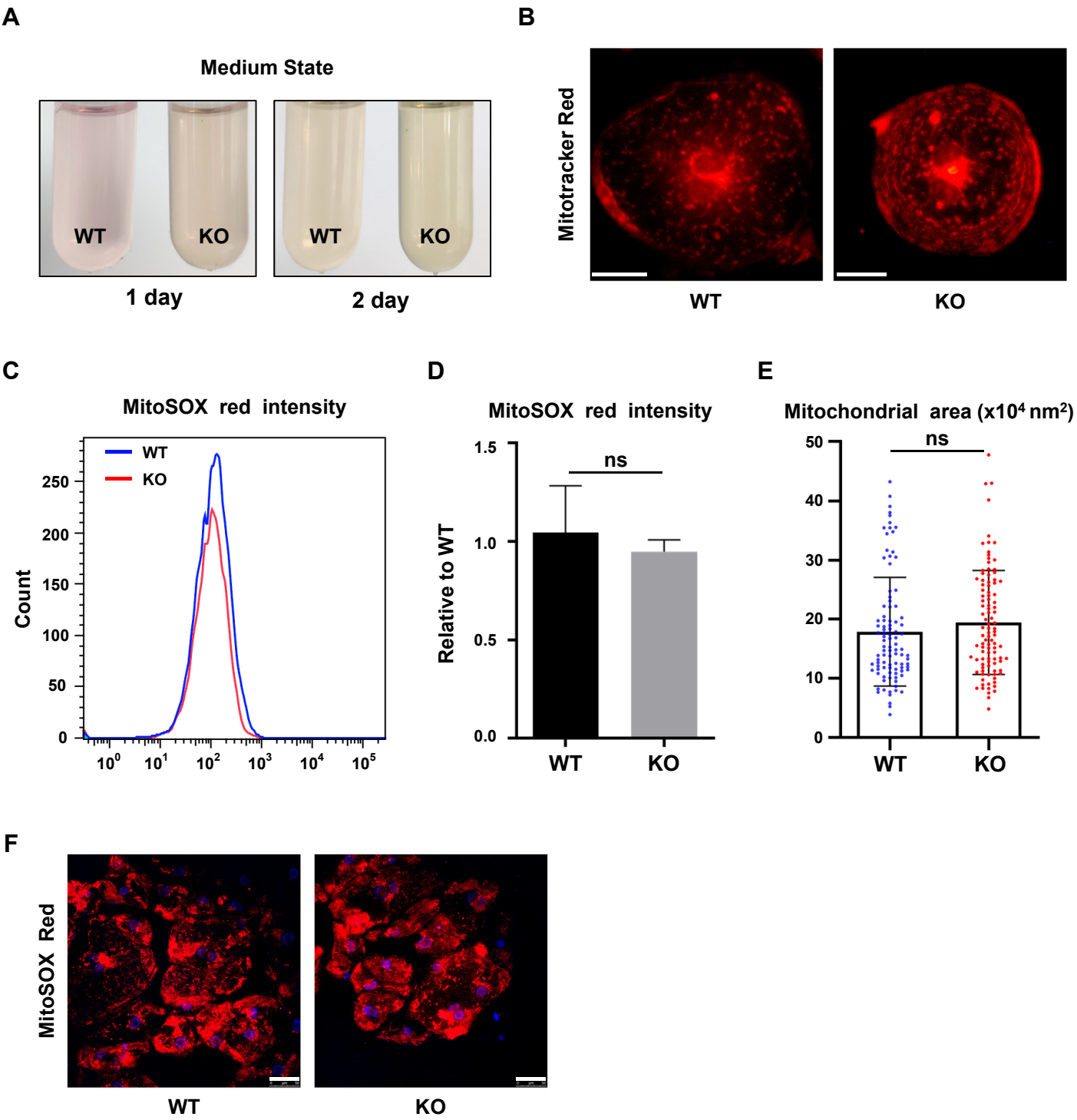

Supplement: Supplementary file 5 — Figure S5 [file JCMM-25-1661-s005.pdf]

FIGURE S6

A

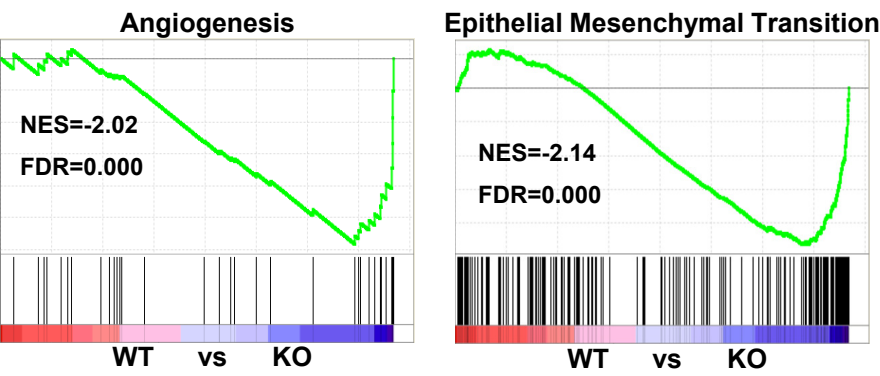

B

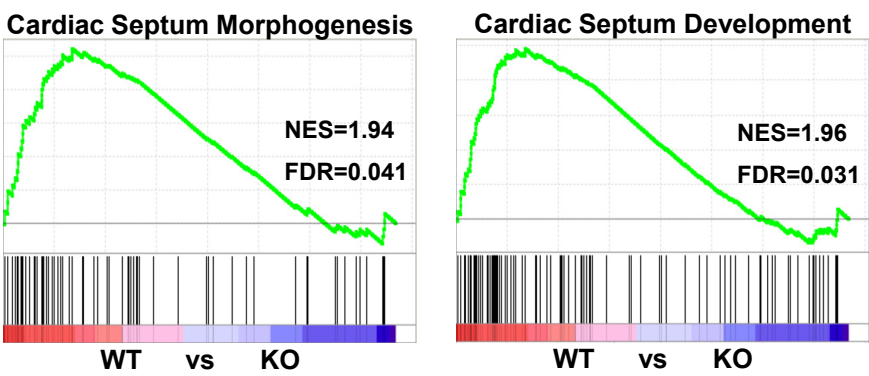

Supplement: Supplementary file 6 — Figure S6 [file JCMM-25-1661-s006.pdf]
