# Supplementary material for: Knockout of MYOM1 in human cardiomyocytes leads to myocardial atrophy via impairing calcium homeostasis
Source: J Cell Mol Med. 2021 Jan 15;25(3):1661–76. doi: 10.1111/jcmm.16268 (PMC7875908; doi:10.1111/jcmm.16268)

FIGURE S7

A

sgRNA PAM

5'···AGAATTAAAGAAGCTGCTGCTTATATAGCCCAGAGGAATCTT··· 3'

3'···TCTTAATTTCTTCGACGACGAATATATCGGGTCTCCTTAGAA··· 5'

#2 AGAATTAAAGAAGCTGCTGCTTATATAGC--AGAGGAATCTT −2bp

#7 AGAATTAAAGAAGCTGCTGCTTATATAGCCCCAGAGGAATCTT +1bp

#12 AGAATTAAAGAAGCTGCTGCTTAT-----CCAGAGGAATCTT −5bp

B

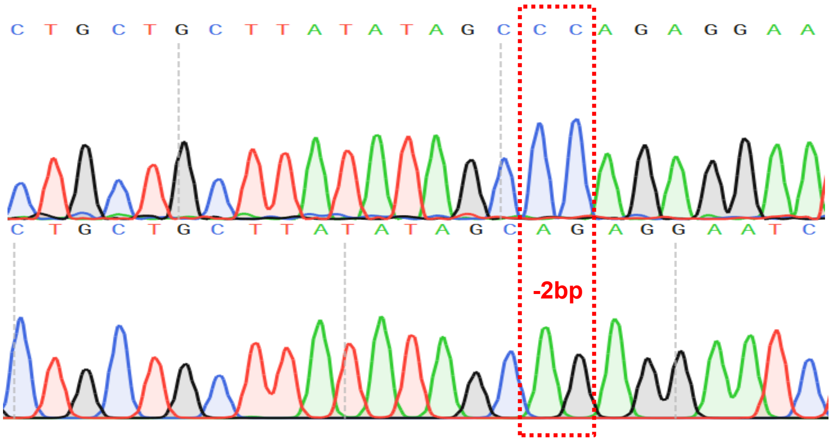

C

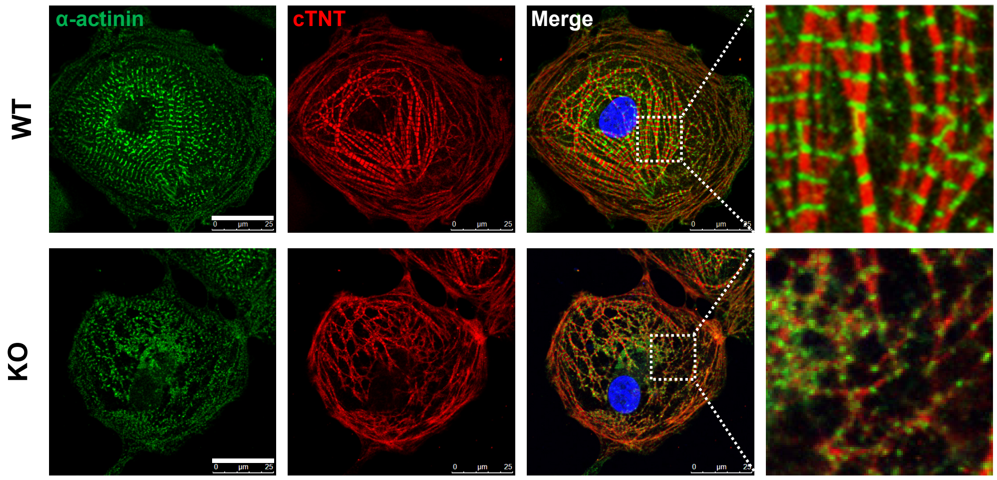

Supplement: Supplementary file 7 — Figure S7 [file JCMM-25-1661-s007.pdf]
