# Supplementary material for: Knockout of MYOM1 in human cardiomyocytes leads to myocardial atrophy via impairing calcium homeostasis
Source: J Cell Mol Med. 2021 Jan 15;25(3):1661–76. doi: 10.1111/jcmm.16268 (PMC7875908; doi:10.1111/jcmm.16268)
Supplement: Supplementary file 8 — Table S1 [file JCMM-25-1661-s008.docx]

Table S1. Primary and Secondary Antibodies

| Type | Antibody | Application | Dilution | Species | Manufacturer And Catalog Number |
| --- | --- | --- | --- | --- | --- |
|  | Anti-SOX2 | Immunofluorescence | 1:100 | Rabbit monoclonal | Abcam ab92494 |
|  | Anti-SSEA4 | Immunofluorescence | 1:100 | Mouse Monoclonal | Santa Cruz sc-21704 |
|  | Anti-MLC2a | Immunofluorescence | 1:100 | Mouse Monoclonal | Santa Cruz sc-365255 |
|  | Anti-MLC2v | Immunofluorescence | 1:100 | Rabbit Polyclonal | Proteintech 10906-1-AP |
|  | Anti-α-actinin | Immunofluorescence | 1:100 | Rabbit Polyclonal | Abcam ab137346 |
|  | Anti-α-actinin | Immunofluorescence | 1:100 | Mouse Monoclonal | Santa Cruz  sc-17829 |
|  | Anti-cTnT | Immunofluorescence | 1:100 | Mouse Monoclonal | Santa Cruz  sc-33721 |
|  | Anti-cTnT | Immunofluorescence | 1:100 | Rabbit Polyclonal | Abcam ab45932 |
|  |  | Flow cytometry | 1:100 |  |  |
|  | Anti-MYOM1 | Immunofluorescence | 1:100 | Rabbit  Monoclonal | Abcam  ab201228 |
|  |  | Western blot | 1:1000 |  |  |
|  | Anti-Cx43 | Immunofluorescence | 1:100 | Rabbit Polyclonal | Cell signaling  #3512S |
|  | Anti-MYH7 | Western blot | 1:1000 | Mouse Monoclonal | Abcam  ab174640 |
|  | Anti-MuRF1 | Western blot | 1:1000 | Mouse Monoclonal | Santa Cruz  sc-398608 |
|  | Anti-MAFbx | Western blot | 1:1000 | Mouse Monoclonal | Santa Cruz  sc-166806 |
|  | Anti-ANP | Western blot | 1:1000 | Rabbit Monoclonal | Abcam  ab209232 |
|  | Anti- p-CaMKII | Western blot | 1:1000 | Mouse  Monoclonal | Santa Cruz  sc-32289 |
|  | Anti-CaMKII | Western blot | 1:1000 | Rabbit Monoclonal | Abcam ab52476 |
|  | Anti- CaMKIIδ | Western blot | 1:1000 | Rabbit Monoclonal | Abcam  ab181052 |
|  | Anti- CaMKIIγ | Western blot | 1:1000 | Mouse  Monoclonal | Abcam  ab201966 |
|  | Anti-Calmodulin | Western blot | 1:1000 | Mouse  Monoclonal | Santa Cruz  sc-137079 |
|  | Anti-Calcineurin | Western blot | 1:1000 | Rabbit Polyclonal | Cell signaling  #2614 |
|  | Anti-GAPDH | Western blot | 1:1000 | Rabbit monoclonal | Abcam  ab181603 |
| Secondary | Goat anti-Mouse IgG Alexa Fluor 594 | Immunofluorescence | 1:200 | Goat anti- Mouse IgG | Invitrogen A21145 |
|  | Goat anti-Rabbit IgG Alexa Fluor 488 | Immunofluorescence | 1:200 | Goat anti- Rabbit IgG | Invitrogen A32731 |
|  | Chicken anti- Rabbit IgG Alexa Fluor 594 | Immunofluorescence | 1:200 | Chicken anti- Rabbit IgG | Invitrogen A21442 |
|  | Chicken anti- Mouse IgG Alexa Fluor 488 | Immunofluorescence | 1:200 | Chicken anti- Mouse IgG | Invitrogen A21200 |
|  | Goat anti-Rabbit IgG (H + L) IRDye 800CW | Western blot | 1:10000 | Goat anti- Rabbit IgG | LI-COR 926-32211 |
|  | Goat anti-Mouse IgG (H + L) IRDye 800CW | Western blot | 1:10000 | Goat anti- Mouse IgG | LI-COR 926-32210 |
