# Supplementary material for: Knockout of MYOM1 in human cardiomyocytes leads to myocardial atrophy via impairing calcium homeostasis
Source: J Cell Mol Med. 2021 Jan 15;25(3):1661–76. doi: 10.1111/jcmm.16268 (PMC7875908; doi:10.1111/jcmm.16268)
Supplement: Supplementary file 9 — Table S2 [file JCMM-25-1661-s009.docx]

Table S2. Primer sequences used for qPCR

| **Gene** | **Forward (5’-3’)** | **Reverse (5’-3’)** |
| --- | --- | --- |
| SSEA4 | TGGACGGGCACAACTTCATC | GGGCAGGTTCTTGGCACTCT |
| SOX2 | ACAGATGCAACCGATGCACC | TGGAGTTGTACTGCAGGGCG |
| OCT4 | CCTGAAGCAGAAGAGGATCACC | AAAGCGGCAGATGGTCGTTTGG |
| NANOG | CTCTCCTCTTCCTTCCTCCAT | TTGCGACACTCTTCTCTGC |
| KLF4 | CATCTCAAGGCACACCTGCGAA | TCGGTCGCATTTTTGGCACTGG |
| LIN28 | CCAGTGGATGTCTTTGTGCACC | GTGACACGGATGGATTCCAGAC |
| FOXD3 | AAGCCGCCTTACTCGTACATCG | AGAGGTTGTGGCGGATGCTGTT |
| CX43 | GGAGATGAGCAGTCTGCCTTTC | TGAGCCAGGTACAAGAGTGTGG |
| ACTB | CACCATTGGCAATGAGCGGTTC | AGGTCTTTGCGGATGTCCACGT |
| ND1 | ATGGCCAACCTCCTACTCCTCATT | TTATGGCGTCAGCGAAGGGTTGTA |
| ND2 | CCATCTTTGCAGGCACACTCATCA | ATTATGGATGCGGTTGCTTGCGTG |
| CSRP3 | CACTGCGAAGTTTGGAGAGTCC | AGCGGAAACAGGTCTTGTGCCA |
| TCAP | GGAAGGATCTGACACTGTCCAC | ATGCCCATCCGCATCATCAGCC |
| Desmin | TCCAGTCCTACACCTGCGAGAT | CGCAATGTTGTCCTGGTAGCCA |
| MYPN | CTCCATCTCCTAAGGTTGAGTGG | CCTCAGCAATGACCAAGGTGCA |
| ACTN2 | GAGGGCAAGATGGTGTCGGATA | CTTCTCAGCCAGGTGTTCCAAG |
| NEBL | GGAATGCAAGCTGGCACTGACA | GAGTGTCTGTGCTCACCTGCAT |
| NRAP | CTTGACCTTCCTGGCAGCCAAA | GGCTCATTTGCGAATCTCCCTG |
| OBSL1 | TCGCTGGAGATGAGTGTGCCTA | TAGCTCACAGGTCAGCACCACA |
| OBSCN | CAGCTCCATTGTCAGGGTGCAT | GGACGTTGTTTCCATAGCACCAC |
| MYOM2 | CTACCTGGACAAGCGTGAAGTTC | CGTAGAGTGAGCCTTCCGTCAA |
| MYOM3 | GCCGATGAAGACATCTCCGCAA | CCAGGATGTCAATGTTCCAGCC |
| MYH6 | TCTCCGACAACGCCTATCAGTAC | GTCACCTATGGCTGCAATGCT |
| MYH7 | GGCAAGACAGTGACCGTGAAG | CGTAGCGATCCTTGAGGTTGTA |
| TTN | CTGCTGACTACACCTTTGTGGC | GCTCGCTTCTTCTCCAGTACCT |
| CAMK2D | ACACGGTGACTCCTGAAGCCAA | GTCTCCTGTCTGTGCATCATGG |
| ANKRD1 | CGACTCCTGATTATGTATGGCGC | GCTTTGGTTCCATTCTGCCAGTG |
| MuRF1 | AAGCCAGTGGTCATCTTGCCGT | CTCCAGACATGGACACTGAGCT |
| CRYAB | ACTTCCCTGAGTCCCTTCTACC | GGAGAAGTGCTTCACATCCAGG |
| FHL1 | ACCGCTGTGGAGGACCAGTATT | AGTGGAAGCAGTAGTCGTGCCA |
| FHL2 | GTGGTGTGCTTTGAGACCCTGT | GAGCAGTGGAAACAGGCTTCATG |
| Erk2 | ACACCAACCTCTCGTACATCGG | TGGCAGTAGGTCTGGTGCTCAA |
| P62 | TGTGTAGCGTCTGCGAGGGAAA | AGTGTCCGTGTTTCACCTTCCG |
| Nbr1 | CCTGAGAGCTTGCTCCAGTCTA | CTTGGTTCCTGGCTGAAGGTGA |
| MAFbx | CACTGGTCCAAAGAGTCGGCAA | GCACAAAGGCAGGTCAGTGAAG |
| CAPN1 | GGTGGAGTTCAACATCCTGTGG | ATCCGCATCTCGTAGGCACTCA |
| BNIP3 | TCAGCATGAGGAACACGAGCGT | GAGGTTGTCAGACGCCTTCCAA |
| FOXO3 | TCTACGAGTGGATGGTGCGTTG | CTCTTGCCAGTTCCCTCATTCTG |
| NPPA | ACAATGCCGTGTCCAACGCAGA | CTTCATTCGGCTCACTGAGCAC |
| NPPB | TCTGGCTGCTTTGGGAGGAAGA | CCTTGTGGAATCAGAAGCAGGTG |
| GAPDH | GGAGCGAGATCCCTCCAAAAT | GGCTGTTGTCATACTTCTCATGG |
